# Structural synaptogenesis superior to functional modulation in a pruning-based recurrent network model of OCD

**DOI:** 10.3389/fncom.2026.1799705

**Published:** 2026-07-20

**Authors:** Ngo Cheung

**Affiliations:** Independent Researcher, Hong Kong, Hong Kong SAR, China

**Keywords:** computational psychiatry, CSTC, GRU, OCD, pruning, SSRI, neurosteroids, ketamine

## Abstract

**Background:**

Obsessive-compulsive disorder (OCD) is characterized by intrusive thoughts and repetitive behaviors, with incomplete response to current treatments suggesting limitations in prevailing neurotransmitter-focused models. Epidemiological data indicate lifetime DSM-IV OCD in approximately 2.3% and 12-month OCD in approximately 1.2% of US adults, while subthreshold obsessions or compulsions are substantially more common. One mechanistic possibility is that abnormal synaptic pruning contributes to persistent cortico-striato-thalamo-cortical circuit rigidity. This idea remains indirect in OCD, but is supported by convergent synaptic-marker findings in OCD and by complement-linked pruning mechanisms shown most clearly in schizophrenia.

**Methods:**

We developed a modular gated recurrent unit network approximating cortico-striato-thalamo-cortical dynamics, trained on a rule-switching task sensitive to perseveration. The architecture included a recurrent cortical integration layer, a schematic striatal “habit” module, and a thalamic confidence-gating scalar. Excessive pruning was implemented as 60% activity-dependent pruning, using a composite of low recent gradient-based usage and low weight magnitude, with partial protection of recurrent and habit-related weights. From identical pruned baselines, three mechanistically distinct interventions were simulated: rapid gradient-guided structural reopening, treated as a ketamine-like synaptogenesis motif, prolonged low-learning-rate adaptation with stress-noise annealing (SSRI-like), and tonic inhibitory scaling (neurosteroid-like). An iso-dose pipeline recorded L1 and L2 weight-change norms, synaptic turnover, and change in sparsity; linear interpolation was used when bracketing sweep points existed, and residual mismatch was reported otherwise. Multi-seed statistics and sensitivity analyses were performed.

**Results:**

At 60% activity-dependent pruning, the untreated network showed impaired accuracy (0.4972) and elevated perseveration (0.5247). In the fixed-parameter comparison, ketamine-like structural repair reduced perseveration to 0.2582, SSRI-like adaptation to 0.3209, and neurosteroid-like inhibition to 0.2654. Relapse vulnerability differed by mechanism: cumulative relapse increased perseveration by +0.1086 after ketamine-like repair, +0.0259 after SSRI-like adaptation, and −0.0017 after neurosteroid-like inhibition in the representative seed. Across five seeds, best acute perseveration was lowest for ketamine-like repair (0.2330 ± 0.0065), followed by neurosteroid-like inhibition (0.2413 ± 0.0021) and SSRI-like adaptation (0.2831 ± 0.0099). SSRI-like adaptation showed the highest mean efficiency because it produced smaller absolute weight changes. Sensitivity analyses showed that untreated perseveration increased with pruning severity, from 0.2627 at 40% pruning to 0.6218 at 70% pruning, while ketamine-like repair remained relatively stable across the same range.

**Conclusion:**

These findings support excessive synaptic pruning as a plausible contributor to OCD-like cognitive inflexibility and illustrate that structural and functional interventions offer different trade-offs within a highly abstract computational model. Structural repair produced the most robust acute rescue and remained resilient across pruning severities, whereas functional mechanisms showed advantages in dose efficiency or relapse stability. The results are hypothesis-generating only and should not be read as clinical evidence for treatment ranking.

## Introduction

Obsessive-compulsive disorder (OCD) is common, with a lifetime prevalence of approximately 2.3% and a 12-month prevalence of approximately 1.2% in the National Comorbidity Survey Replication (Ruscio et al., 2010). More than one quarter of respondents in that survey reported obsessions or compulsions at some point, suggesting that the public health burden of obsessive-compulsive symptoms may be broader than categorical prevalence alone implies (Ruscio et al., 2010). The disorder’s mix of intrusive thoughts and repetitive rituals can erode work, study, and relationships. Standard care—exposure-based cognitive-behavioural therapy or a selective serotonin re-uptake inhibitor—helps many sufferers, yet full remission is the exception rather than the rule; when treatment stops, relapse is frequent and residual symptoms linger for many patients (Fineberg et al., 2012; Pallanti and Quercioli, 2006).

For years, most biological accounts have centred on hyperactive cortico-striato-thalamo-cortical loops and on disturbed glutamate, serotonin, and dopamine signalling within those networks (Milad and Rauch, 2012; Pittenger et al., 2011). These ideas inspired useful symptom-based treatments, but they do not fully explain why the disorder often begins in childhood or why medicines that target the main transmitters leave many patients only partially improved.

A new thread has come up recently. Current research on synaptic markers suggests that altered excitatory connectivity may be relevant to OCD, including lower excitatory synaptic gene expression in orbitofrontal cortex and striatum and lower synaptic density associated with cognitive dysfunction (Piantadosi et al., 2021; Xiao et al., 2024). The strongest mechanistic genetic example for complement-linked synaptic pruning comes from schizophrenia rather than OCD: structurally diverse complement component 4 alleles influence C4A expression, human C4 localizes to synaptic compartments, and C4 mediates synapse elimination in mice (Sekar et al., 2016). These findings do not prove complement-mediated over-pruning in OCD, but they provide a biologically plausible template for testing how pruning disturbances might alter cortico-striatal computations.

Computational models let researchers test such mechanistic stories in silico. Computational psychiatry explicitly combines theory-driven models with data-driven approaches to improve mechanistic understanding, prediction, and treatment selection in mental illness (Huys et al., 2016). Earlier work has already linked OCD to faulty reinforcement learning, oversized uncertainty estimates, and habit dominance (Huys et al., 2016; Maia and Frank, 2011). Formal reinforcement-learning accounts have framed psychiatric symptoms in terms of abnormal prediction errors, action valuation, and policy updating (Maia and Frank, 2011). In OCD specifically, dimensional work has linked compulsive symptoms to impaired goal-directed control and relative habit dominance (Gillan et al., 2016), while predictive-inference models have proposed that obsessions and compulsions may reflect an attempt to find stable anchors in an uncertain environment (Fradkin et al., 2020). Related studies have examined altered information gathering and decision thresholds in juvenile OCD (Hauser et al., 2017), the psychometric behavior of predictive-inference tasks (Loosen et al., 2024), and basal-ganglia models of OCD symptoms and deep brain stimulation (Tewari et al., 2017). Together, these models have clarified several functional computations, but they have rarely asked whether abnormal synapse elimination itself can create the circuit conditions for perseveration. What is missing is a model that weaves abnormal pruning into circuit dynamics and compares, on equal footing, treatments that rebuild structure with those that tune activity. The study introduced below fills that gap. Using a recurrent network that mimics cortico-striatal interactions, we ask whether activity-dependent synaptic loss can generate perseveration in a rule-switching task, and whether structural regrowth, slow functional adaptation, and tonic inhibition show separable advantages under a transparent dose-matching procedure. The resulting predictions are intended to guide hypotheses, not clinical decisions.

## Methods

### Network architecture and CSTC circuit approximation

**Figure 1:**
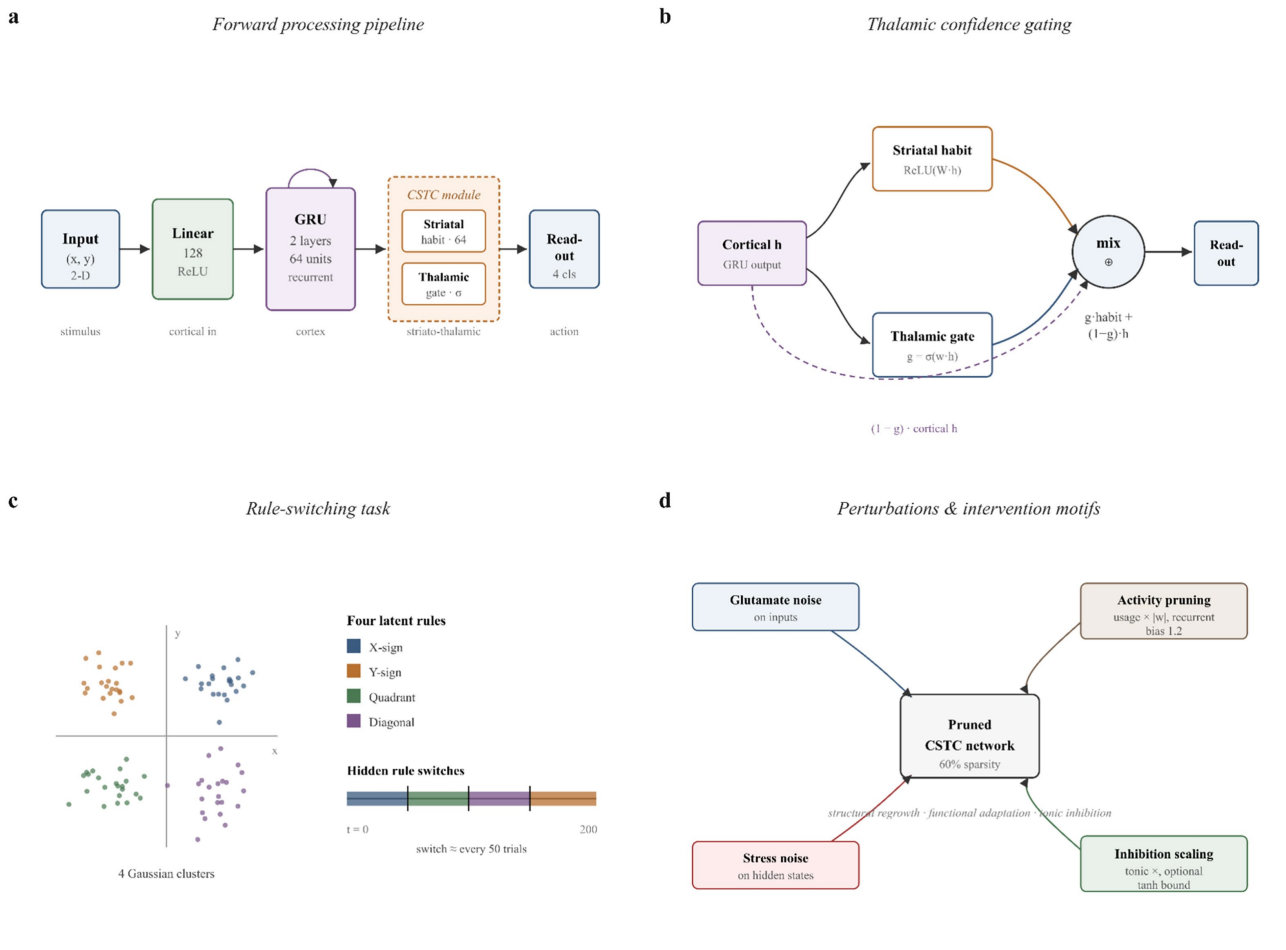
Computational architecture of the cortico-striato-thalamo-cortical (CSTC) loop model. **(a)** Forward processing pipeline. A two-dimensional stimulus is projected by a 128-unit ReLU linear layer into a two-layer gated recurrent unit (GRU, 64 hidden units per layer) approximating recurrent cortical integration, followed by a modular striato-thalamic stage and a four-class linear read-out. **(b)** Thalamic confidence gating. The recurrent cortical state drives a striatal “habit” transformation and a sigmoidal thalamic gate g; the action representation is a gated mixture, g·habit + (1 − g)·cortical state, providing a schematic goal-directed versus habitual balance. **(c)** Rule-switching task. Two-dimensional points are drawn from four Gaussian clusters and labelled by four latent rule labels implemented in code as X-sign, Y-sign, quadrant, and diagonal mappings; because the Y-sign and quadrant formulae are algebraically equivalent in the present implementation, the effective task contains three distinct response mappings rather than four fully independent mappings. The active rule label switches at hidden change-points approximately every 50 trials within 200-trial sequences. **(d)** Perturbation and intervention motifs acting on the network: hidden-state stress noise, activity-dependent (“complement-like”) pruning combining recent gradient-based usage with weight magnitude under a protective recurrent bias, and tonic inhibitory activation scaling. All components are deliberate abstractions and are not pathway-level biological models.

All simulations were run in PyTorch (Figure 1). Inputs (two features) passed through a 128-unit linear layer with ReLU activation, into a two-layer gated recurrent unit (GRU; 64 hidden units each, 0.1 dropout), and finally to a four-class linear read-out. A GRU is a gated recurrent neural network unit designed to carry information across a sequence while deciding how much of the previous hidden state should be retained or overwritten (Cho et al., 2014). In this model, the GRU state was used as a compact proxy for recurrent cortical task-set maintenance rather than as a literal population of neurons. Schematically, following the PyTorch convention, the GRU update can be written in text form as:


r_t=sigmoid(W_irx_t+b_ir+W_hrh_(t−1)+b_hr)

z_t=sigmoid(W_izx_t+b_iz+W_hzh_(t−1)+b_hz)

$$n\_t=\tanh(\begin{array}{l} W\_\text{in}\;x\_t+b\_\text{in}+r\_t\;\text{elementwise}\_\text{multiply}\\ \;(W\_\mathrm{hn}\;h\_(t-1)+b\_\mathrm{hn}) \end{array})$$

$$\begin{array}{l} h\_t=(1-z\_t)\;\text{elementwise}\_\text{multiply}\;n\_t+\\ z\_t\;\text{elementwise}\_\text{multiply}\;h\_(t-1) \end{array}$$

Here, r_t is the reset gate, z_t is the update gate, n_t is the candidate hidden state, h_t is the updated recurrent state, sigmoid is the logistic sigmoid function, and elementwise_multiply denotes element-wise multiplication. In plain terms, the GRU can preserve a representation of the recent rule context while allowing that representation to change when the task changes. This role is close to the computational idea of recurrent prefrontal activity supporting working memory and task-set maintenance, although the model is much simpler than a biological prefrontal circuit (Miller and Cohen, 2001; Miller et al., 2003).

To provide a more explicit cortico-striato-thalamo-cortical approximation, the recurrent output also passed through a schematic striatal “habit” module and a thalamic confidence gate. The striatal module was implemented as a 64-unit linear transformation with ReLU activation. The thalamic gate was implemented as a sigmoid scalar that mixed habit-module activity with recurrent cortical activity before action selection. In text equation form, the habit representation was H_t = ReLU(W_s h_t + b_s), the thalamic confidence gate was g_t = sigmoid(W_g h_t + b_g), and the mixed action representation was m_t = g_t elementwise_multiply H_t + (1−g_t) elementwise_multiply h_t. The final read-out mapped m_t to four response logits.

The sensory layer therefore approximated early stimulus encoding; the GRU approximated recurrent cortical integration and task-set memory; the habit module approximated striatal action bias; and the gate approximated thalamic or cortico-thalamic confidence gating within a broad CSTC motif (Milad and Rauch, 2012; Alexander et al., 1986). The mapping is functional rather than anatomical. The model does not contain direct and indirect basal ganglia pathways, realistic thalamic relay dynamics, cell classes, or neurotransmitter-specific synapses. Its value is that each component has a clear computational role that can be perturbed and compared under controlled conditions.

Recurrent and habit-related weights were treated as partially protected during pruning to approximate persistence of recurrent loop structure. The model class included both input-level Gaussian noise and hidden-state stress noise. In the reported treatment simulations, hidden-state stress noise was used for SSRI-like adaptation and relapse modeling; task difficulty analyses varied stimulus-generation noise. The explicit glutamate-noise parameter was implemented in the code but was not activated in the main reported experiments. A multiplicative scalar applied to hidden activations represented tonic neurosteroid-like inhibition; optional tanh bounding was used in the neurosteroid-like condition.

### Cognitive flexibility task

Set-shifting impairment and perseverative responding are well-described neuropsychological findings in OCD and related compulsive phenotypes (Chamberlain et al., 2005; Chamberlain et al., 2006). To test set shifting, the model learned a rule-switch task modeled after the Wisconsin Card Sorting Test. The network had to infer which hidden rule label was currently active from training experience and then change its response policy when the rule label changed without an explicit external cue. Thus, the task was not designed to model obsessions or rituals directly. It was designed to isolate flexible rule updating.

Each stimulus was a two-dimensional point drawn from four Gaussian clusters (centres ±1.5, SD 0.8). Labels were assigned by one of four possible rule labels: X-sign, Y-sign, quadrant, or diagonal. These rule labels were chosen because they use the same simple two-dimensional stimulus space but require different response mappings in principle. In the present code, however, the Y-sign and quadrant formulae are algebraically equivalent, so the effective task contains three distinct mappings. The task is therefore best interpreted as a controlled hidden-rule-label switching task, not as a fully balanced four-rule WCST analogue. Sequences contained 200 trials, with the active rule switching roughly every 50 trials after an initial burn-in. Training used 400 randomly generated sequences; testing used 100 fixed sequences with deterministic switches. Batch size was 32. Perseverative errors immediately after a hidden rule switch were treated as the main computational phenotype because they capture a narrow but measurable form of cognitive rigidity: repeating a recent response when the environment now requires updating.

### Baseline training and developmental pruning

Networks were first trained from scratch for 40 epochs with Adam (learning rate 0.001, gradient-clip 1.0) to reach a healthy baseline. To model excessive developmental pruning, 60% of weights were removed using an activity-dependent rule rather than 95% magnitude pruning. A very severe 95% magnitude-pruning setting was considered during exploratory model development, but it was not used as the main pruning condition because it behaved more like an extreme lesion and depended only on weight size. The 60% activity-dependent setting was chosen as a more interpretable stress test: it was severe enough to impair set shifting, but not so severe that the network was reduced to a near-disconnected system.

Recent usage was estimated by accumulating absolute gradients over training batches. A composite importance score was computed from gradient-based usage and absolute weight magnitude; synapses with low recent usage and low magnitude were preferentially removed. In practical terms, gradient-based usage asked whether changing a weight had recently mattered for task learning, while weight magnitude asked whether the connection had become numerically strong. A synapse was therefore most vulnerable when it was both weak and recently uninformative for the task. This is still a machine-learning rule, not a biological pruning law. It was used because it is closer to activity-dependent refinement than pure magnitude pruning, which removes small weights without considering recent function.

The biological motivation is indirect. Complement-mediated synaptic refinement has been shown most clearly in developmental and disease-relevant neuroscience, including work showing that the classical complement cascade can mediate synapse elimination in the central nervous system (Stevens et al., 2007) and that structurally diverse C4 alleles influence schizophrenia risk through effects on C4A expression and synapse elimination (Sekar et al., 2016). In OCD, the evidence is much less direct, but lower excitatory synaptic gene expression in orbitofrontal cortex and striatum and lower synaptic density associated with cognitive dysfunction suggest that altered synaptic architecture may be relevant to at least some patients (Piantadosi et al., 2021; Xiao et al., 2024). The present pruning rule was therefore intended as a computational proxy for activity-dependent synaptic loss, not as a model of microglial biology. It does not simulate microglial motility, C1q/C3/C4 tagging, cell-type specificity, local cytokine states, developmental timing, dendritic compartments, or human synaptic-density imaging.

**Figure 2:**
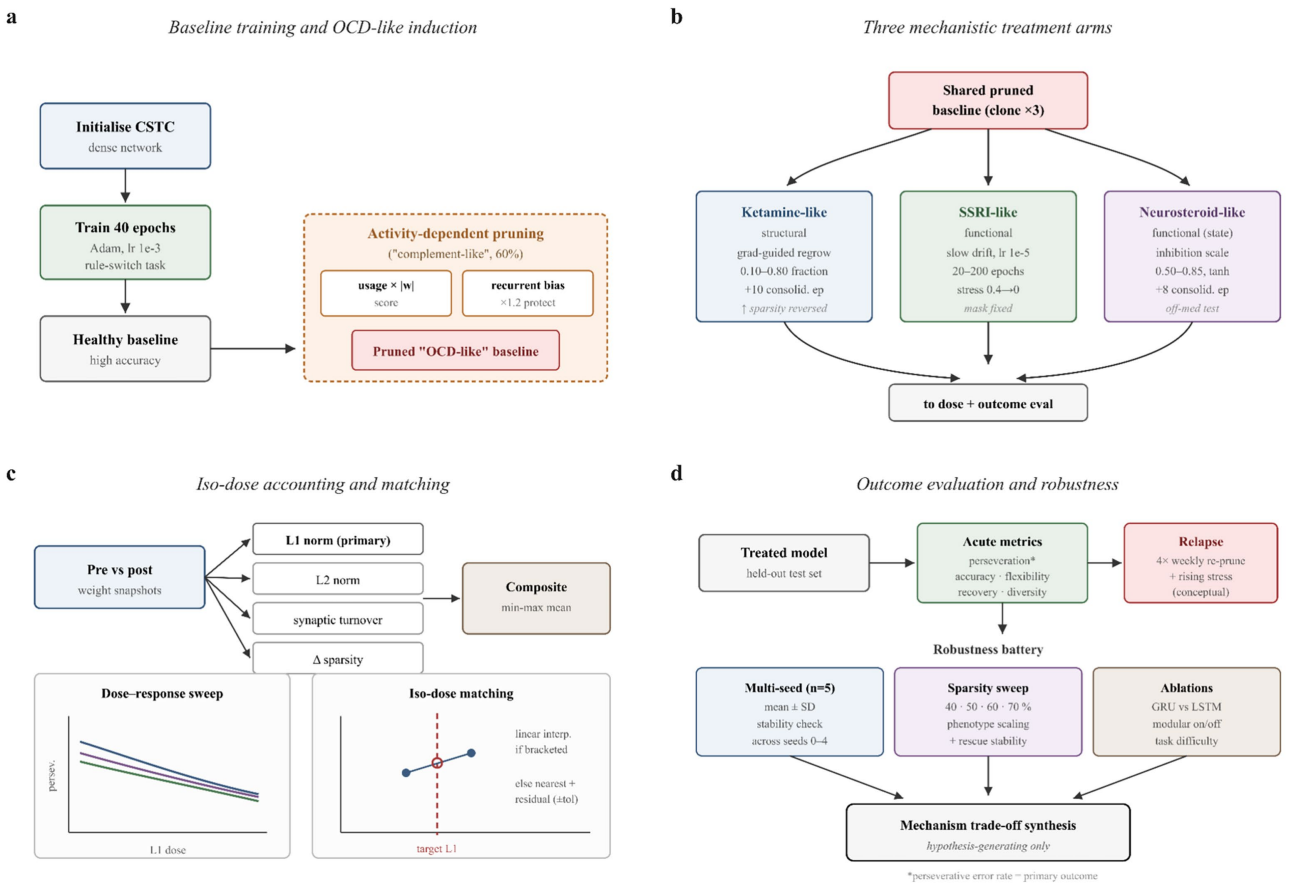
Experimental pipeline. **(a)** A dense CSTC network is initialised and trained for 40 epochs on the rule-switching task to a healthy baseline, after which an activity-dependent (“complement-like”) rule removes 60% of weights using a composite of recent gradient-based usage and weight magnitude, with a protective recurrent bias, yielding a pruned “OCD-like” baseline. **(b)** The shared pruned baseline is cloned three times and exposed to mechanistically distinct interventions: ketamine-like structural gradient-guided mask reopening and consolidation, SSRI-like slow low-learning-rate adaptation with stress-noise annealing under a fixed mask, and neurosteroid-like tonic inhibitory scaling tested both on- and off-medication. Each arm is run as a single representative setting and as a parameter sweep. **(c)** For every treated model, pre- and post-treatment weight snapshots yield four dose descriptors (L1 norm as primary, plus L2 norm, synaptic turnover and change in sparsity) and a min-max composite; sweeps build dose–response curves, and iso-dose matching uses linear interpolation between bracketing points or reports the nearest configuration with its residual mismatch against a fixed tolerance. **(d)** Treated models are scored on acute set-shifting metrics (perseverative error rate as the primary outcome) and on a conceptual cumulative relapse procedure, then subjected to a robustness battery comprising multi-seed statistics, pruning-severity sweeps and architectural/task ablations, feeding a mechanism trade-off synthesis that is hypothesis-generating only.

Recurrent and habit-related weights received a protective bias of 1.2 (Figure 2). This bias modestly increased the importance score of recurrent and habit-related parameters before pruning, making them slightly less likely to be removed. It was included to approximate the idea that recurrent loop structure and overlearned action pathways may persist despite widespread synaptic refinement. The value 1.2 was not fitted to biological data; it was a conservative modeling choice, and an ablation without recurrent protection was included to test whether the main result depended on it.

This pruning rule is a computational proxy for activity-dependent, complement-like synaptic refinement. It does not simulate microglia, C1q/C3/C4 signalling, cell-type-specific tagging, or a true developmental time course. The resulting sparsified networks reliably showed increased perseveration. Pruning masks stayed in place for all later steps unless growth was explicitly triggered.

### Treatment mechanism simulations

Three treatments started from identical pruned models. The three arms were chosen to contrast broad intervention motifs rather than to simulate medications at receptor or pharmacokinetic level. The names “ketamine-like,” “SSRI-like,” and “neurosteroid-like” therefore refer to abstract computational mechanisms: structural capacity reopening, slow functional adaptation, and tonic inhibitory stabilization.

Ketamine-like: gradient-guided structural reopening restored mask access to 60% of previously pruned weights in the fixed-parameter comparison, followed by 10 consolidation epochs at learning rate 0.0005. Parameter sweeps tested regrowth fractions from 0.10 to 0.80. The mechanism was inspired by rapid plasticity and by proof-of-concept ketamine findings in OCD, but no receptor-level pharmacology was modeled (Rodriguez et al., 2013). The biological motivation was the literature linking NMDA-antagonist-related rapid antidepressant effects to synaptic plasticity, including mTOR-dependent synapse formation and downstream plasticity mechanisms (Li et al., 2010; Duman et al., 2012; Kavalali and Monteggia, 2012). In the model, however, this arm simply reopened previously pruned parameter masks selected by gradient information and allowed those parameters to be retrained. Because treatment arms were cloned from the stored pruned baseline, the operation should be read as structural capacity reopening plus consolidation, not as exact recovery of original dense pre-pruning weights and not as a claim that ketamine restores a specific set of lost synapses in OCD patients.

SSRI-like: networks continued training for 120 epochs at a learning rate of 1e-5 while stress noise was linearly reduced from 0.4 to zero; the pruning mask remained fixed. Parameter sweeps tested 20 to 200 additional epochs. This mechanism represented slow functional adaptation rather than serotonin receptor pharmacodynamics. The motivation was the clinical pattern that SSRIs are established treatments for OCD but usually act over weeks and often leave residual symptoms (Fineberg et al., 2012; Soomro et al., 2008). A meta-analysis of fixed-dose SSRI trials in adults with OCD found that higher SSRI doses were associated with greater efficacy, although this was partly counterbalanced by more dropouts due to side effects (Bloch et al., 2010). In the present arm, no new connections were created. Instead, the model was allowed to make small weight adjustments while a stress-like noise term was gradually reduced. This stress-noise term is only a computational nuisance variable; it does not represent cortisol, autonomic arousal, inflammation, or any single biological stress pathway, although stress is known to affect synaptic and glutamatergic function more broadly (Popoli et al., 2011).

Neurosteroid-like: hidden activations were scaled by 0.65 in the fixed-parameter comparison, with tanh bounding enabled, and the model was trained for eight consolidation epochs. Parameter sweeps tested inhibition strengths from 0.50 to 0.85. During off-medication tests, the scaling factor was removed. This arm was motivated by the role of neurosteroids in modulating GABAergic inhibition, including tonic and extrasynaptic GABA-A receptor effects (Farrant and Nusser, 2005; Herd et al., 2007; Zorumski et al., 2019). In the model, tonic inhibition was represented only as multiplicative scaling of hidden activity. It did not include receptor subtypes, endogenous steroid synthesis, pharmacokinetics, sedation, network oscillations, or region-specific inhibitory interneurons. The off-medication test asked whether the behavioral benefit persisted when this scaling was removed; it did not simulate withdrawal biology.

### Relapse simulation

Relapse was modeled conceptually. In the fixed-parameter comparison, relapse consisted of four cumulative “weekly” steps of recurrent-biased secondary pruning, with each step removing 12% of active non-recurrent weights and applying a 1.5-fold pruning multiplier to recurrent and striatal weights, together with a small stress increase. In the dose–response sweeps, relapse was tested with a single 40% secondary pruning step to preserve comparability across sweep points. These procedures are illustrative devices and do not model clinical relapse biology. They were intended to test whether each intervention left the network vulnerable to a renewed structural or stress-like perturbation. This is narrower than clinical relapse, which may involve learning history, stress exposure, medication discontinuation, avoidance, expectancy, and return of fear or threat associations (Vervliet et al., 2013).

### OCD-relevant evaluation metrics

Performance on the held-out test set was summarised by (1) overall accuracy, (2) perseverative error rate—operationalized as the fraction of incorrect responses within the 10-trial post-switch window in which the model repeated its immediately preceding response, (3) flexibility index, defined as early post-switch accuracy divided by stable pre-switch accuracy, (4) repetition rate, (5) output diversity, and (6) trials-to-recovery. The perseverative error rate was the primary outcome. This metric does not prove that the model is repeating the previous latent rule; it measures immediate response repetition after rule-label change.

### Iso-dose fair comparison pipeline and dose definition

To compare mechanisms on equal footing, dose was defined primarily as the mean L1 change in weights, computed as absolute weight change divided by parameter count. For a model with N trainable parameters, the primary L1 dose was defined in text form as:


$$\begin{array}{l} D\_L1={\left(1/N\right)}^{*}\;\mathrm{sum}\;\text{over}\;i=1\;\text{to}\;N\;\text{of absolute}\_\text{value}\\ (w\_i\_\text{post}-w\_i\_\mathrm{pre}) \end{array}$$

Here, w_i_pre is the value of parameter i before treatment and w_i_post is its value after treatment. In simple terms, this number asks how much the model’s weights changed on average. L1 was chosen because it is transparent, easy to interpret, and less dominated by a small number of large changes than a squared metric. Weight norms are standard tools for describing model parameters and perturbations in machine learning, but here they are used only as within-model accounting devices (Bishop, 2006; Goodfellow et al., 2016).

Additional descriptors included L2 change, synaptic turnover, defined as the fraction of weights whose relative change exceeded 10% of their pre-treatment magnitude, and net sparsity shift. A composite dose score was also computed by min-max scaling L1, L2, turnover, and sparsity change and averaging the four quantities. L2 change was included to capture larger concentrated shifts, turnover to capture how many weights changed meaningfully, and sparsity shift to capture whether a treatment actually changed the number of active connections. These descriptors were reported because structural reopening and functional scaling are not truly equivalent operations.

Parameter sweeps produced dose–response curves: regrow fraction 0.10–0.80 for ketamine-like, 20–200 extra epochs for SSRI-like, and inhibition strength 0.50–0.85 for neurosteroid-like. For target L1 doses, linear interpolation was used only when the target was bracketed by two achieved sweep points. When a target lay outside a treatment’s achieved range, the nearest achieved configuration was reported with its residual mismatch and whether that mismatch was within the pre-specified tolerance of 0.002. Efficiency was reported as perseveration reduction per unit L1 change. The main single-seed experiments used random seed 42. For the neurosteroid-like condition, L1 dose captured only treatment-associated weight changes during consolidation and did not directly quantify the instantaneous activation-scaling factor. The iso-dose procedure should therefore be read as a computational fairness device, similar to comparing interventions after matching the amount of model-state displacement. It is not a pharmacological dose, not receptor occupancy, not plasma concentration, not exposure duration, and not a measure of tolerability or adverse effects (Adams et al., 2016).

### Modeling choices and their rationale

Activity-dependent pruning was chosen because pure magnitude pruning removes weak weights without considering recent task use. The implemented score therefore combined recent gradient-based usage with weight magnitude. This remains a simplified proxy: biological synaptic pruning depends on cell type, molecular tags, developmental stage, local activity, immune signalling, and glial state.

The L1 iso-dose metric was chosen as a transparent computational convenience. It is not a pharmacological dose, not receptor occupancy, not plasma concentration, and not a measure of clinical exposure. It allows within-model comparison of how much network state changed, but structural regrowth and functional scaling are not truly commensurable on one scalar.

A multi-seed analysis was performed with five seeds. Sensitivity analyses tested pruning severity from 40 to 70%, GRU versus LSTM recurrence, modular versus non-modular architecture, removal of recurrent protective bias, and task difficulty manipulations.

## Results

### Induction of an OCD-like phenotype through activity-dependent pruning

The trained network learned the rule-switching task before pruning. After 60% activity-dependent pruning, the untreated model showed an OCD-like rigidity profile: accuracy was 0.4972, perseverative error rate was 0.5247, and flexibility index was 0.9717. In qualitative terms, the pruned network was more likely to repeat the previous response after hidden rule switches, reproducing a narrow form of set-shifting rigidity. This should not be taken as a full OCD phenotype, since the task does not model intrusive threat, affective salience, checking, contamination fear, or relief-driven rituals.

### Comparison of three treatments at representative settings

We next applied one representative parameter set per treatment to the same pruned baseline (Table 1).

**Table 1:** Multi-mechanism treatment comparison at fixed parameters.

Treatment	L1 dose	Turnover	Δ Sparsity	Acute accuracy (Δ)	Acute perseveration (Δ)	Acute flexibility (Δ)	Efficiency	Relapse Δ perseveration
Untreated	—	—	—	0.4972	0.5247	0.9717	—	—
Ketamine-like	0.008160	0.5113	0.3345	0.7435 (+0.2463)	0.2582 (−0.2665)	0.9726 (+0.0009)	32.66	+0.1086
SSRI-like	0.003245	0.1605	0.0000	0.7183 (+0.2211)	0.3209 (−0.2038)	0.9752 (+0.0035)	62.83	+0.0259
Neurosteroid- like	0.006393	0.2473	0.0000	0.7318 (+0.2346)	0.2654 (−0.2593)	0.9716 (−0.0001)	40.56	−0.0017

Δ values are relative to the untreated baseline. Efficiency = perseveration reduction divided by L1 dose.

Ketamine-like rapid structural reopening produced the lowest acute perseveration in the fixed comparison, reducing perseveration from 0.5247 to 0.2582. It also produced the largest structural change, with L1 dose 0.008160, turnover 0.5113, and change in sparsity 0.3345. After cumulative relapse, perseveration rose by +0.1086, indicating that structural benefit was substantial but not immune to renewed pruning.

SSRI-like slow adaptation produced smaller absolute acute improvement but the highest efficiency, because the L1 dose was lower. Perseveration fell from 0.5247 to 0.3209, with L1 dose 0.003245 and no change in sparsity. After cumulative relapse, perseveration increased by +0.0259.

Neurosteroid-like inhibition produced an acute perseveration value close to the ketamine-like condition in the representative seed, falling to 0.2654, and showed the smallest relapse rebound in this fixed comparison. Because the mechanism depends on continued functional scaling, its interpretation differs from structural regrowth: benefit may reflect medication-state stabilization rather than repair of the pruned network.

### Dose–response and iso-dose matching

Across parameter sweeps, the three mechanisms occupied partly overlapping but not identical L1 dose ranges (Tables 2, 3).

**Table 2:** Dose–response summary across parameter sweeps, single seed.

Treatment	Achieved L1 range	Best acute perseveration	Best relapse *Δ*	Max efficiency	Mean efficiency
Ketamine-like	0.005782–0.009680	0.2588	+0.0316	45.4384	36.4385
SSRI-like	0.000965–0.004252	0.2930	−0.0097	107.8521	72.2164
Neurosteroid-like	0.005860–0.007200	0.2613	−0.0242	43.3534	40.5222

**Table 3:** Iso-dose matching at target L1 dose 0.005, single seed.

Treatment	Nearest parameter	Actual L1	Residual mismatch	Within tolerance	Acute perseveration
Ketamine-like	regrow_fraction = 0.10	0.005782	0.000782	True	0.2620
SSRI-like	epochs = 200	0.004252	0.000748	True	0.2930
Neurosteroid-like	strength = 0.85	0.005860	0.000860	True	0.2707

The 0.010 target used in earlier exploratory runs was not retained as a formal iso-dose target because the achieved sweep range ended at L1 = 0.009680. At the target L1 dose of 0.005, exact bracketing was not available for every treatment, so nearest achieved configurations and residual mismatches were reported.

At this approximate iso-dose point, ketamine-like repair produced the lowest acute perseveration, neurosteroid-like inhibition was close behind, and SSRI-like adaptation showed a smaller acute effect but remained within the dose tolerance. The comparison should be interpreted cautiously because exact interpolation was not possible across all mechanisms at this target.

### Multi-seed statistics

To test whether the single-seed pattern was stable, the experiment was repeated over five seeds (Table 4).

**Table 4:** Multi-seed statistics, *n* = 5.

Metric	Ketamine-like, mean ± SD	SSRI-like, mean ± SD	Neurosteroid-like, mean ± SD
Best acute perseveration	0.2330 ± 0.0065	0.2831 ± 0.0099	0.2413 ± 0.0021
Mean efficiency	35.2688 ± 8.1057	68.0495 ± 18.1388	36.4710 ± 8.4551
Best relapse Δ	+0.0799 ± 0.0379	+0.0221 ± 0.0459	−0.0159 ± 0.0094

Untreated perseveration across the five seeds was 0.5094 ± 0.0549. Ketamine-like repair showed the lowest best acute perseveration, SSRI-like adaptation showed the highest mean efficiency, and neurosteroid-like inhibition showed the most favorable relapse-delta profile. This pattern supports a trade-off rather than a single universal winner.

### Sensitivity and ablation analyses

Pruning severity strongly affected the untreated phenotype (Table 5). As sparsity increased from 40 to 70%, untreated perseveration increased from 0.2627 to 0.6218. Ketamine-like repair remained comparatively stable across this range, while SSRI-like and neurosteroid-like outcomes deteriorated more at the highest pruning level.

**Table 5:** Pruning sparsity sensitivity.

Pruning sparsity	Untreated perseveration	Ketamine best acute	SSRI best acute	Neurosteroid best acute
0.40	0.2627	0.2596	0.2623	0.2613
0.50	0.3476	0.2604	0.2655	0.2599
0.60	0.5247	0.2588	0.2930	0.2613
0.70	0.6218	0.2604	0.3631	0.3054

Architecture ablations showed that the modular GRU was not the sole source of the effect (Table 6). Removing the modular striatal-thalamic components or switching to LSTM altered untreated perseveration and efficiency, but ketamine-like rescue of acute perseveration persisted. Removing recurrent protective bias had little effect in this configuration.

**Table 6:** Architecture and mechanism ablations.

Architecture/Mechanism	Untreated perseveration	Ketamine best acute	Ketamine mean efficiency
GRU + modular	0.5247	0.2588	36.44
GRU, no modular module	0.5043	0.2521	30.08
LSTM + modular	0.4815	0.2570	20.52
GRU + modular, no recurrent bias	0.5247	0.2588	36.44

Task difficulty also mattered. Longer switch intervals and higher input noise changed perseveration, confirming that the pruning phenotype depends partly on the behavioral environment rather than on sparsity alone (Table 7).

**Table 7:** Task difficulty sensitivity.

Switch interval	Input noise	Perseveration	Flexibility
30	0.8	0.4845	1.0383
50	0.8	0.4938	1.0375
50	1.2	0.5496	0.9744
80	0.8	0.6914	0.9872

## Discussion

### Interpretation of the computational findings

**Figure 3:**
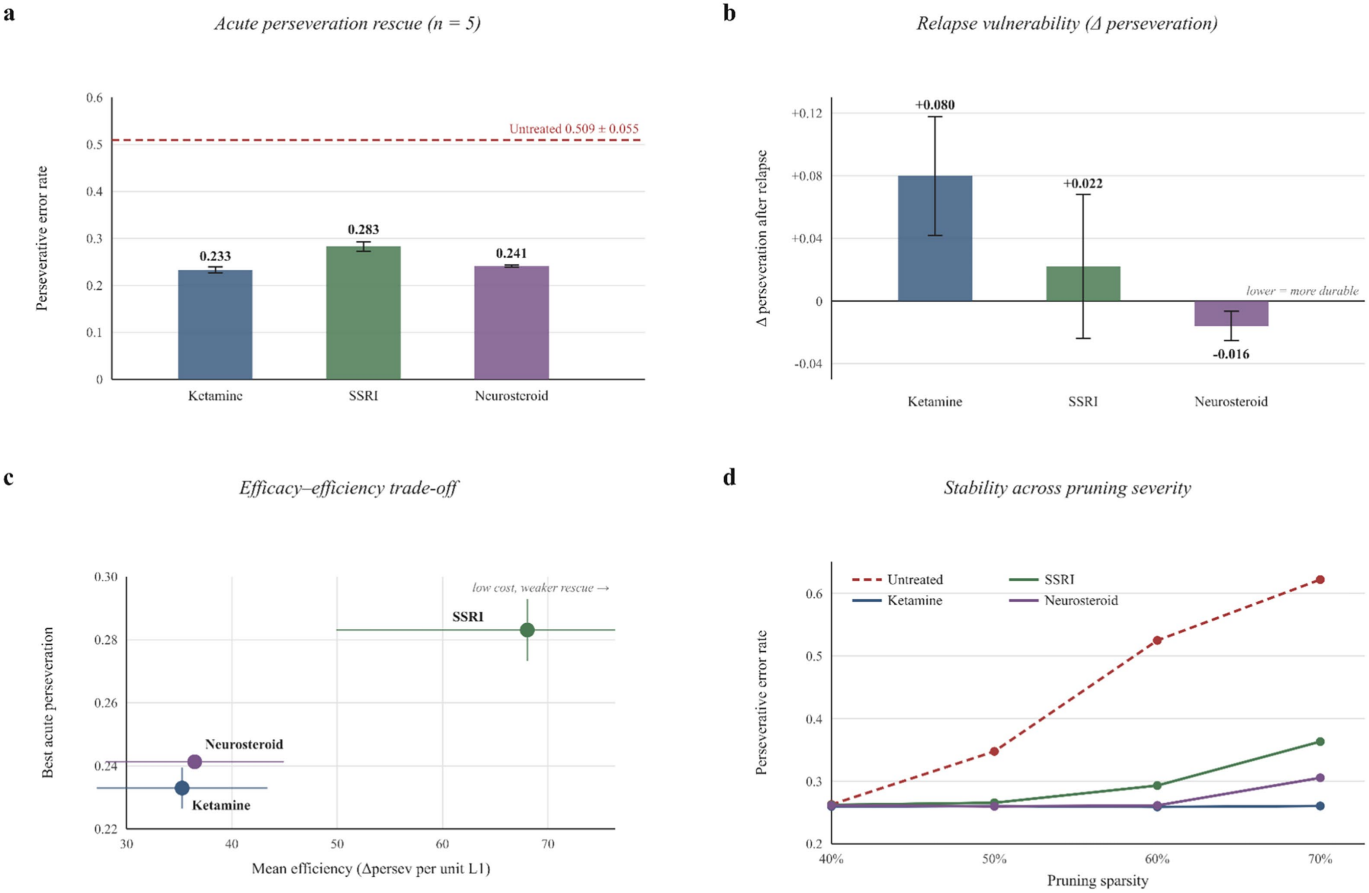
Interpretation of the computational findings. Treatment effects are summarised from the multi-seed analysis (*n* = 5; mean ± s.d.) and the pruning-severity sweep. **(a)** Best acute perseverative error rate for each mechanism relative to the untreated pruned baseline (dashed red, 0.509 ± 0.055). Ketamine-like structural repair achieved the lowest acute perseveration (0.233 ± 0.007), with neurosteroid-like inhibition close behind (0.241 ± 0.002) and SSRI-like adaptation showing a smaller acute effect (0.283 ± 0.010). **(b)** Change in perseveration after the conceptual relapse procedure; positive values indicate rebound. Ketamine-like repair showed the largest rebound (+0.080 ± 0.038), whereas neurosteroid-like inhibition was the most durable (−0.016 ± 0.009). **(c)** Efficacy–efficiency trade-off, plotting best acute perseveration against mean dose efficiency (perseveration reduction per unit L1 weight change). SSRI-like adaptation occupied a high-efficiency, weaker-rescue regime because it altered weights least, while structural repair achieved stronger rescue at greater network cost. **(d)** Robustness across pruning severity (40–70%). Untreated perseveration rose steeply with sparsity, whereas ketamine-like repair remained essentially flat; SSRI- and neurosteroid-like outcomes degraded mainly at the most severe pruning level. Together the panels illustrate a mechanism-dependent trade-off rather than a single universal winner. All results are hypothesis-generating within a highly abstract model and are not clinical evidence.

Our simulations support the idea that excessive synaptic pruning can contribute to OCD-like cognitive inflexibility in a rule-switching setting (Figure 3). When 60% of weights were removed by an activity-dependent rule, the model became less accurate and more perseverative. This was not a complete model of OCD, but it reproduced one measurable feature of the disorder: difficulty shifting away from a previously reinforced rule.

Among the three interventions, ketamine-like structural repair produced the strongest acute rescue in multi-seed testing and remained stable across pruning-severity sensitivity analyses. This supports the limited conclusion that, within this model, rebuilding pruned connections is particularly effective when the underlying perturbation is structural sparsity. However, the results do not support a simple statement that structural repair is superior in every respect. SSRI-like adaptation showed higher dose efficiency because it changed weights less, and neurosteroid-like inhibition showed the most favorable relapse-delta profile in the multi-seed summary.

The SSRI-like schedule led to meaningful but smaller acute improvement. In the model, no new synapses appeared; noise was tapered while weights drifted slowly. This mirrors, at a high level, the clinical observation that selective serotonin re-uptake inhibitors help many people but often require prolonged treatment and may leave residual symptoms (Fineberg et al., 2012; Soomro et al., 2008). The adult OCD dose–response meta-analysis by Bloch and colleagues further suggests that higher SSRI doses can improve efficacy, but at the cost of greater side-effect-related dropout (Bloch et al., 2010). The model does not simulate serotonin receptors, downstream intracellular signalling, dose escalation, adverse effects, or exposure-based learning, so this comparison remains schematic.

The neurosteroid-like condition produced rapid functional stabilization by scaling hidden activity. Its acute effect was close to the ketamine-like condition in the representative seed, and its relapse-delta profile was favorable. Still, this mechanism was medication-state dependent in the model: removing the scaling factor tests a different question from removing newly regrown weights. For that reason, neurosteroid-like benefit should be interpreted as functional stabilization rather than structural repair.

Ketamine’s advantage in the model is consistent with, but not validated by, early clinical data. In the proof-of-concept randomized crossover trial in OCD, drug-free adults with near-constant obsessions received 40-min intravenous infusions of saline and ketamine 0.5 mg/kg, separated by at least one week; carryover effects complicated the crossover analysis, and first-phase data showed that 50% of those receiving ketamine met Y-BOCS response criteria one week later compared with 0% receiving placebo (Rodriguez et al., 2013). The synaptogenic interpretation is also broadly consistent with preclinical work linking NMDA-antagonist-related rapid behavioral effects to mTOR-dependent synapse formation and other plasticity mechanisms (Li et al., 2010; Duman et al., 2012; Kavalali and Monteggia, 2012). That study was small and proof-of-concept. The present model should therefore be read as a mechanistic hypothesis about why rapid plasticity might matter, not as clinical evidence that ketamine should outrank established OCD treatments.

### Speculative clinical implications and therapeutic priorities

The modelling work may help frame future treatment hypotheses, but it should not be used to alter clinical guidelines on its own (Table 8). Selective serotonin re-uptake inhibitors and exposure-based cognitive-behavioural therapy remain established treatments (Fineberg et al., 2012; Soomro et al., 2008). The computational result suggests that patients whose symptoms are driven by structural circuit under-connectivity might require different strategies from patients whose symptoms are mainly maintained by state-dependent noise, anxiety, or inhibitory imbalance. This is a stratification hypothesis, not a clinical recommendation.

**Table 8:** Speculative therapeutic implications derived from the pruning-centric computational model.

Therapeutic intervention	Mechanism and model insight	Clinical status and limitations	Hypothesis-generating priority
SSRIs	Functional adaptation: reduces stress-like noise and permits gradual weight drift without directly restoring pruned connections.	Established pharmacological treatment for OCD, often requiring weeks to months; response is incomplete in many patients (Fineberg et al., 2012; Gillan et al., 2016).	Retain as standard care; investigate whether pruning-related biomarkers predict slower or incomplete response.
Synaptogenic agents, including ketamine-like mechanisms	Structural repair: regrows pruned weights and directly addresses sparsity in the model.	Early OCD evidence is limited to small proof-of-concept work; ketamine is not established as routine OCD treatment (Hauser et al., 2017).	Test as a mechanistic candidate in carefully designed trials, especially in refractory or biologically stratified groups.
Neurosteroid-like inhibition	Tonic functional stabilization: dampens activity and improves performance without changing sparsity.	Neurosteroid evidence is not OCD-specific; benefit may be state-dependent (Fradkin et al., 2020; Cho et al., 2014).	Study as a possible adjunctive stabilizer rather than as a proven stand-alone treatment for core OCD circuitry.
Consolidation strategies, including CBT or stimulation	Stabilization: may protect newly adaptive circuit states after plasticity induction.	Exposure therapy is established; stimulation approaches require careful indication and protocol selection (Tewari et al., 2017).	Test whether behavioral or neuromodulatory consolidation prolongs benefit after rapid plasticity interventions.
Precision stratification	Etiology matching: links treatment mechanism to hypothesized pruning, stress-noise, or inhibition-dominant phenotypes.	Complement-pruning evidence is strongest in schizophrenia, and direct OCD biomarkers remain underdeveloped (Sekar et al., 2016).	Develop biomarkers cautiously; avoid assuming that complement or microglial markers already define treatment choice in OCD.

The simulations also suggest how treatment might be consolidated. Networks that underwent regrowth still remained vulnerable to renewed pruning, mirroring the clinical concern that rapid benefits may fade without follow-up care. Combining plasticity-promoting interventions with exposure-based therapy, booster sessions, or plasticity-inducing brain stimulation could be worth testing (Carmi et al., 2019). This is a research hypothesis only.

Finally, a pruning framework opens the door to precision prescribing, but the evidence is not yet sufficient. Patients with high polygenic burden in complement or microglial pathways might eventually form one biologically meaningful subgroup, but this remains speculative. At present, complement C4 data provide a strong example of immune-linked pruning biology in schizophrenia, not a validated OCD treatment-selection marker (Sekar et al., 2016).

### Novelty and potential impact

**Figure 4:**
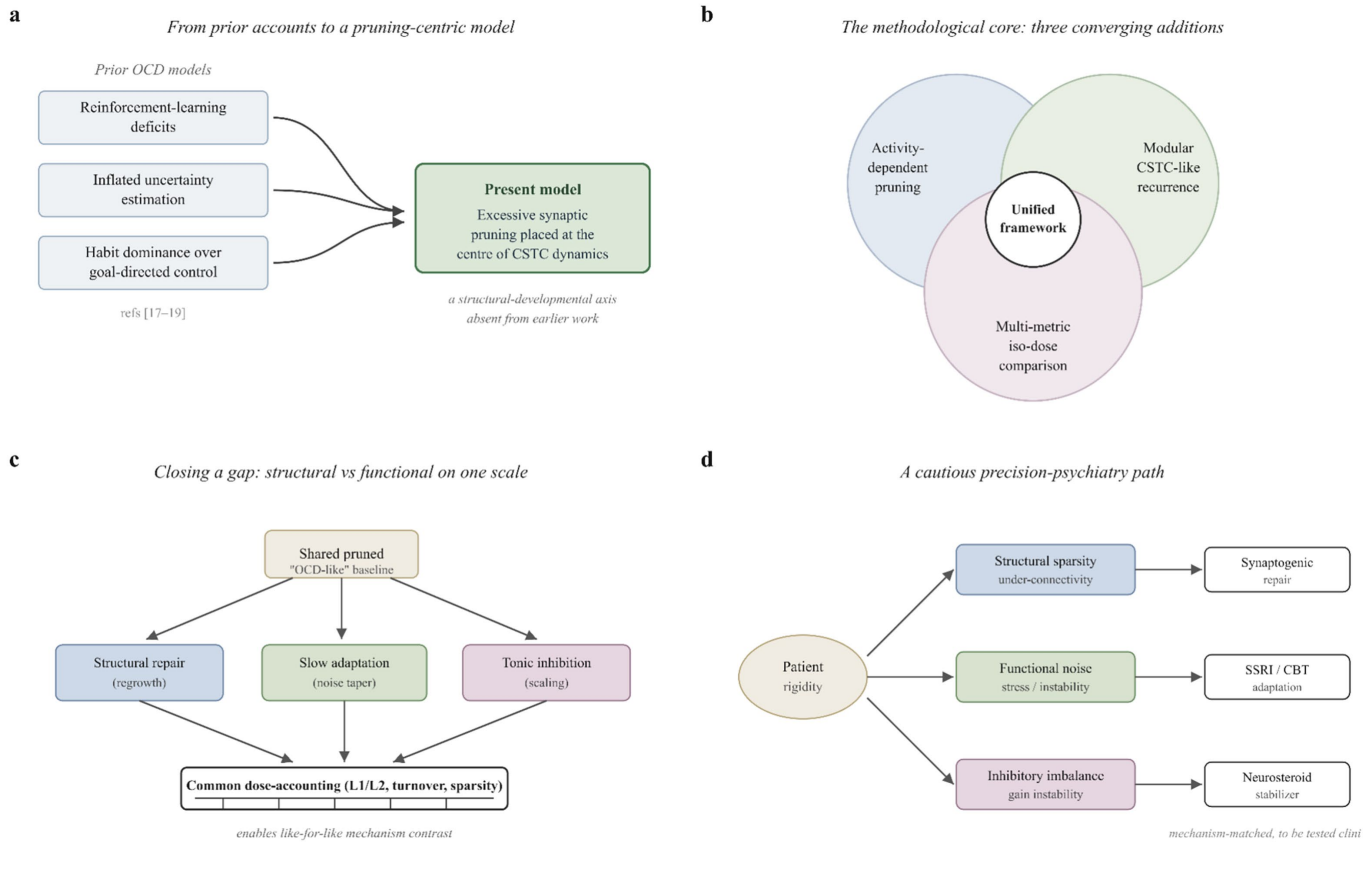
Novelty and potential impact of the pruning-centric framework. **(a)** Conceptual positioning. Earlier computational accounts of OCD emphasised reinforcement-learning deficits, inflated uncertainty, altered decision thresholds, and habit dominance (Gillan et al., 2016; Fradkin et al., 2020; Hauser et al., 2017; Loosen et al., 2024; Tewari et al., 2017); the present work instead places exaggerated, activity-dependent synaptic pruning at the centre of cortico-striato-thalamo-cortical (CSTC) dynamics, adding a structural-developmental axis largely absent from prior models. **(b)** Methodological core. The contribution is the integration of three elements that are rarely combined: activity-dependent (“complement-like”) pruning, a modular CSTC-like recurrent architecture, and a multi-metric iso-dose comparison, which together form a single transparent framework within theory-driven computational psychiatry. **(c)** Closing a methodological gap. From one shared pruned baseline, structural repair, slow functional adaptation, and tonic inhibition are placed on a common dose-accounting ruler (L1/L2 weight change, synaptic turnover, sparsity shift), enabling a like-for-like contrast between structural and functional interventions that previous studies did not provide. **(d)** Potential translational impact. The framework motivates a cautious precision-psychiatry path: determine whether an individual’s rigidity is best explained by structural sparsity, functional noise, or inhibitory imbalance, then prospectively test mechanism-matched interventions. This panel depicts a hypothesis-generating workflow only and does not assert validated clinical decision rules.

The present work is, to our knowledge, among the first to place exaggerated developmental pruning at centre stage in a recurrent model of cortico-striato-thalamo-cortical-like circuitry and then weigh competing treatment motifs after recording the magnitude of network change (Figure 4). Earlier OCD simulations have concentrated on skewed reinforcement learning, inflated uncertainty, altered decision thresholds, or habit dominance (Gillan et al., 2016; Fradkin et al., 2020; Hauser et al., 2017; Loosen et al., 2024; Tewari et al., 2017). They rarely modelled synapse elimination or contrasted structural and functional interventions under a shared dose-accounting framework.

The contribution is methodological rather than clinically prescriptive. The model combines activity-dependent pruning, modular recurrent architecture, multi-metric dose accounting, residual mismatch reporting, multi-seed statistics, and sensitivity analyses. This fits within the theory-driven branch of computational psychiatry, where explicit mechanistic assumptions are made transparent and then tested against simulated behavior (Huys et al., 2016; Adams et al., 2016).

### Modeling assumptions and limitations

This is a hypothesis-generating in-silico exploration, not a validated mechanistic or clinical model. Every result should be read through this lens.

First, pruning is a computational proxy. The model uses activity-dependent weak-synapse pruning at 40–70% sparsity to stand in for synaptic loss. It does not replicate microglia, complement signalling, developmental timing, molecular tagging, or human imaging measures of synaptic density.

Second, CSTC fidelity is limited. The striatal habit module and thalamic confidence gate are crude abstractions. The model lacks pathway-specific neurochemistry, direct and indirect basal ganglia pathways, cell-type specificity, orbitofrontal subregions, and realistic thalamocortical dynamics. Likewise, the GRU gates are mathematical operations for retaining or updating a hidden state; they are not ion channels, synaptic receptors, or anatomically separated cortical cell populations.

Third, iso-dose is not pharmacological dose. L1 and L2 weight-change norms, synaptic turnover, and sparsity change quantify network alteration. They are not receptor occupancy, plasma concentration, clinical dose, exposure, tolerability, or adverse-effect burden. Structural and functional changes are not fully commensurable on one scalar. In particular, the L1 metric does not directly count the neurosteroid-like activation scaling itself, only treatment-associated weight changes during consolidation.

Fourth, the task-phenotype mapping is narrow. Rule-switching captures set-shifting rigidity only. It omits intrusive threat, affective dysregulation, disgust, checking, reassurance seeking, harm avoidance, relief learning, and the personal meaning of obsessions. The optional ritual-action variant is conceptual and was not used as the primary result. The implemented rule set also contains four latent rule labels but only three distinct effective response mappings because two sign-based mappings are algebraically equivalent in the code.

Fifth, relapse is conceptual. Cumulative recurrence-biased pruning with rising stress is an illustrative device, not a biologically grounded relapse model.

Sixth, clinical implications are speculative. Any apparent treatment ordering holds only within this abstraction. The model does not show that ketamine, SSRIs, neurosteroids, brain stimulation, or psychotherapy should be ranked differently in real patients. No patient data calibrated these simulations.

Seventh, the multi-seed sample remains small. Five seeds provide a more honest estimate than a single run, but they do not cover the full space of architectures, tasks, hyperparameters, or patient heterogeneity.

### Concluding remarks

Despite these constraints, the study supports a neuro-developmental hypothesis in which excessive synaptic pruning can produce a narrow form of OCD-like cognitive inflexibility. Structural repair showed the most robust acute rescue and the greatest stability across pruning severities, while functional mechanisms showed distinct advantages in efficiency and relapse stability. Coupling these insights with biomarkers—such as pruning-related polygenic scores or longitudinal synaptic imaging—and with carefully controlled trials of rapid plasticity interventions could move the field toward more mechanism-guided hypotheses. The present findings should be treated as a computational starting point, not as clinical proof.

## Data Availability

Publicly available datasets were analyzed in this study. This data can be found at: https://github.com/cheungngo/Structural-Synaptogenesis-Superior-to-Functional-Modulation-in-a-Network-Model-of-OCD.

## References

[ref1] Adams R. A. Huys Q. J. M. Roiser J. P. (2016). Computational psychiatry: towards a mathematically informed understanding of mental illness. J. Neurol. Neurosurg. Psychiatry 87, 53–63. doi: 10.1136/jnnp-2015-310737, 26157034 PMC4717449

[ref2] Alexander G. E. DeLong M. R. Strick P. L. (1986). Parallel organization of functionally segregated circuits linking basal ganglia and cortex. Annu. Rev. Neurosci. 9, 357–381. doi: 10.1146/annurev.ne.09.030186.002041, 3085570

[ref3] Bishop C. M. (2006). Pattern Recognition and Machine Learning. New York: Springer.

[ref4] Bloch M. H. McGuire J. Landeros-Weisenberger A. Leckman J. F. Pittenger C. (2010). Meta-analysis of the dose-response relationship of SSRI in obsessive-compulsive disorder. Mol. Psychiatry 15, 850–855. doi: 10.1038/mp.2009.50, 19468281 PMC2888928

[ref5] Carmi L. Tendler A. Bystritsky A. Hollander E. Blumberger D. M. Daskalakis J. . (2019). Efficacy and safety of deep transcranial magnetic stimulation for obsessive-compulsive disorder: a prospective multicenter randomized double-blind placebo-controlled trial. Am. J. Psychiatry 176, 931–938. doi: 10.1176/appi.ajp.2019.18101180, 31109199

[ref6] Chamberlain S. R. Blackwell A. D. Fineberg N. A. Robbins T. W. Sahakian B. J. (2005). The neuropsychology of obsessive compulsive disorder: the importance of failures in cognitive and behavioural inhibition as candidate endophenotypic markers. Neurosci. Biobehav. Rev. 29, 399–419. doi: 10.1016/j.neubiorev.2004.11.006, 15820546

[ref7] Chamberlain S. R. Fineberg N. A. Blackwell A. D. Robbins T. W. Sahakian B. J. (2006). Motor inhibition and cognitive flexibility in obsessive-compulsive disorder and trichotillomania. Am. J. Psychiatry 163, 1282–1284. doi: 10.1176/ajp.2006.163.7.1282, 16816237

[ref8] Cho K. van Merriënboer B. Gulcehre C. Bahdanau D. Bougares F. Schwenk H. ., (2014) Learning phrase representations using RNN encoder-decoder for statistical machine translation Proceedings of the 2014 Conference on Empirical Methods in Natural Language Processing 1724–1734

[ref9] Duman R. S. Li N. Liu R. J. Duric V. Aghajanian G. (2012). Signaling pathways underlying the rapid antidepressant actions of ketamine. Neuropharmacology 62, 35–41. doi: 10.1016/j.neuropharm.2011.08.044, 21907221 PMC3195863

[ref10] Farrant M. Nusser Z. (2005). Variations on an inhibitory theme: phasic and tonic activation of GABA-A receptors. Nat. Rev. Neurosci. 6, 215–229. doi: 10.1038/nrn1625, 15738957

[ref11] Fineberg N. A. Brown A. Reghunandanan S. Pampaloni I. (2012). Evidence-based pharmacotherapy of obsessive-compulsive disorder. Int. J. Neuropsychopharmacol. 15, 1173–1191. doi: 10.1017/S1461145711001829 22226028

[ref12] Fradkin I. Adams R. A. Parr T. Roiser J. P. Huppert J. D. (2020). Searching for an anchor in an unpredictable world: a computational model of obsessive compulsive disorder. Psychol. Rev. 127, 672–699. doi: 10.1037/rev0000188, 32105115

[ref13] Gillan C. M. Kosinski M. Whelan R. Phelps E. A. Daw N. D. (2016). Characterizing a psychiatric symptom dimension related to deficits in goal-directed control. eLife 5:e11305. doi: 10.7554/eLife.11305, 26928075 PMC4786435

[ref14] Goodfellow I. Bengio Y. Courville A. (2016). Deep Learning. Cambridge, MA: MIT Press.

[ref15] Hauser T. U. Moutoussis M. Iannaccone R. Brem S. Walitza S. Drechsler R. . (2017). Increased decision thresholds enhance information gathering performance in juvenile obsessive-compulsive disorder. Biol. Psychiatry 82, 361–369. doi: 10.1371/journal.pcbi.1005440, 28403139 PMC5406001

[ref16] Herd M. B. Belelli D. Lambert J. J. (2007). Neurosteroid modulation of synaptic and extrasynaptic GABA-A receptors. Pharmacol. Ther. 116, 20–34. doi: 10.1016/j.pharmthera.2007.03.007 17531325

[ref17] Huys Q. J. M. Maia T. V. Frank M. J. (2016). Computational psychiatry as a bridge from neuroscience to clinical applications. Nat. Neurosci. 19, 404–413. doi: 10.1038/nn.4238, 26906507 PMC5443409

[ref18] Kavalali E. T. Monteggia L. M. (2012). Synaptic mechanisms underlying rapid antidepressant action of ketamine. Am. J. Psychiatry 169, 1150–1156. doi: 10.1176/appi.ajp.2012.12040531, 23534055

[ref19] Li N. Lee B. Liu R. J. Banasr M. Dwyer J. M. Iwata M. . (2010). mTOR-dependent synapse formation underlies the rapid antidepressant effects of NMDA antagonists. Science 329, 959–964. doi: 10.1126/science.1190287, 20724638 PMC3116441

[ref20] Loosen A. M. Seow T. X. F. Hauser T. U. (2024). Consistency within change: evaluating the psychometric properties of a widely used predictive-inference task. Behav. Res. Methods 56, 7410–7426. doi: 10.3758/s13428-024-02427-y 38844601 PMC11362202

[ref21] Maia T. V. Frank M. J. (2011). From reinforcement learning models to psychiatric and neurological disorders. Nat. Neurosci. 14, 154–162. doi: 10.1038/nn.2723, 21270784 PMC4408000

[ref22] Milad M. R. Rauch S. L. (2012). Obsessive-compulsive disorder: beyond segregated cortico-striatal pathways. Trends Cogn. Sci. 16, 43–51. doi: 10.1016/j.tics.2011.11.003, 22138231 PMC4955838

[ref23] Miller P. Brody C. D. Romo R. Wang X. J. (2003). A recurrent network model of somatosensory parametric working memory in the prefrontal cortex. Cereb. Cortex 13, 1208–1218. doi: 10.1093/cercor/bhg101 14576212 PMC4632206

[ref24] Miller E. K. Cohen J. D. (2001). An integrative theory of prefrontal cortex function. Annu. Rev. Neurosci. 24, 167–202. doi: 10.1146/annurev.neuro.24.1.167, 11283309

[ref25] Pallanti S. Quercioli L. (2006). Treatment-refractory obsessive-compulsive disorder: methodological issues, operational definitions and therapeutic lines. Prog. Neuro-Psychopharmacol. Biol. Psychiatry 30, 400–412. doi: 10.1016/j.pnpbp.2005.11.028, 16503369

[ref26] Piantadosi S. C. Chamberlain B. L. Glausier J. R. Lewis D. A. Ahmari S. E. Sweet R. A. (2021). Lower excitatory synaptic gene expression in orbitofrontal cortex and striatum in an initial study of subjects with obsessive compulsive disorder. Mol. Psychiatry 26, 986–998. doi: 10.1038/s41380-019-0431-3, 31168067

[ref27] Pittenger C. Bloch M. H. Williams K. (2011). Glutamate abnormalities in obsessive compulsive disorder: neurobiology, pathophysiology, and treatment. Pharmacol. Ther. 132, 314–332. doi: 10.1016/j.pharmthera.2011.09.006, 21963369 PMC3205262

[ref28] Popoli M. Yan Z. McEwen B. S. Sanacora G. (2011). The stressed synapse: the impact of stress and glucocorticoids on glutamate transmission. Nat. Rev. Neurosci. 13, 22–37. doi: 10.1038/nrn3138, 22127301 PMC3645314

[ref29] Rodriguez C. I. Kegeles L. S. Levinson A. Feng T. Marcus S. M. Vermes D. . (2013). Randomized controlled crossover trial of ketamine in obsessive-compulsive disorder: proof-of-concept. Neuropsychopharmacology 38, 2475–2483. doi: 10.1038/npp.2013.150, 23783065 PMC3799067

[ref30] Ruscio A. M. Stein D. J. Chiu W. T. Kessler R. C. (2010). The epidemiology of obsessive-compulsive disorder in the National Comorbidity Survey Replication. Mol. Psychiatry 15, 53–63. doi: 10.1038/mp.2008.94, 18725912 PMC2797569

[ref31] Sekar A. Bialas A. R. de Rivera H. Davis A. Hammond T. R. Kamitaki N. . (2016). Schizophrenia risk from complex variation of complement component 4. Nature 530, 177–183. doi: 10.1038/nature16549, 26814963 PMC4752392

[ref32] Soomro G. M. Altman D. Rajagopal S. Oakley-Browne M. (2008). Selective serotonin re-uptake inhibitors versus placebo for obsessive compulsive disorder. Cochrane Database Syst. Rev. 1:CD001765. doi: 10.1002/14651858.CD001765.pub3 PMC7025764 18253995

[ref33] Stevens B. Allen N. J. Vazquez L. E. Howell G. R. Christopherson K. S. Nouri N. . (2007). The classical complement cascade mediates CNS synapse elimination. Cell 131, 1164–1178. doi: 10.1016/j.cell.2007.10.036, 18083105

[ref34] Tewari S. Moustafa A. A. Giegling I. (2017). A neurocomputational model of obsessive-compulsive disorder symptoms and deep brain stimulation. Front. Comput. Neurosci. 11:47.28638335

[ref35] Vervliet B. Craske M. G. Hermans D. (2013). Fear extinction and relapse: state of the art. Annu. Rev. Clin. Psychol. 9, 215–248. doi: 10.1146/annurev-clinpsy-050212-185542, 23537484

[ref36] Xiao Q. Hou J. Xiao L. Zhang W. Zhang Y. Li Y. . (2024). Lower synaptic density and its association with cognitive dysfunction in patients with obsessive-compulsive disorder. Gen. Psychiatry 37:e101208. doi: 10.1136/gpsych-2023-101208 PMC11184172 38894874

[ref37] Zorumski C. F. Paul S. M. Covey D. F. Mennerick S. (2019). Neurosteroids as novel antidepressants and anxiolytics: GABA-A receptors and beyond. Neurobiol. Stress 11:100196. doi: 10.1016/j.ynstr.2019.100196, 31649968 PMC6804800

