# Supplementary material for: Structural synaptogenesis superior to functional modulation in a pruning-based recurrent network model of OCD
Source: Front Comput Neurosci. 2026 Jul 20;20:1799705. doi: 10.3389/fncom.2026.1799705 (PMC13429669; doi:10.3389/fncom.2026.1799705)
Supplement: Supplementary file 1 [file Data_Sheet_1.PDF]

Appendix A. Simulation Results

Computational Model of OCD-like Rigidity, v4.0 (Biologically-Grounded Revision). PyTorch 2.11.0+cu128; CUDA enabled. All values are dimensionless model metrics. Results are hypothesis-generating in-silico estimates and are not clinically calibrated.

A.1 Multi-Mechanism Comparison

Condition	Accuracy	Perseveration	Flexibility	Relapse Δ
Untreated	0.4972	0.5247	0.9717	—
Ketamine (acute)	0.7435	0.2582	0.9726	+0.1086
SSRI (acute)	0.7183	0.3209	0.9752	+0.0259
Neurosteroid (acute)	0.7318	0.2654	0.9716	−0.0017

Note. Baseline pruning mode = activity-dependent; achieved sparsity = 60.0%. Neurosteroid off-medication reversal = +0.0083. Relapse Δ is the change in perseveration under cumulative, recurrence-biased re-pruning.

Treatment	L1 Dose	Turnover	Acute Persev.	Efficiency	Relapse Δ
Ketamine	0.008160	0.5113	0.2582	32.66	+0.1086
SSRI	0.003245	0.1605	0.3209	62.83	+0.0259
Neurosteroid	0.006393	0.2473	0.2654	40.56	−0.0017

A.2 Iso-Dose Dose–Response Sweeps

Param	L1	Turnover	Composite	Acute Persev.	Relapse Δ
Ketamine (regrow fraction)					
0.1	0.005782	0.2677	0.4613	0.2620	+0.0316
0.2	0.006089	0.3095	0.5167	0.2588	+0.0994
0.3	0.006544	0.3591	0.5848	0.2609	+0.2093
0.4	0.006995	0.4085	0.6531	0.2612	+0.2013
0.5	0.007528	0.4575	0.7261	0.2615	+0.2312
0.6	0.008149	0.5098	0.8053	0.2624	+0.2034
0.7	0.008838	0.5626	0.8881	0.2615	+0.2183
0.8	0.009680	0.6172	0.9802	0.2591	+0.2022
SSRI (epochs)					
20	0.000965	0.0184	0.0000	0.4206	+0.1470
40	0.001768	0.0596	0.0670	0.3682	+0.0965

60	0.002278	0.0932	0.1137	0.3548	+0.0894
80	0.002660	0.1212	0.1507	0.3472	+0.0346
100	0.002971	0.1426	0.1809	0.3359	+0.0245
120	0.003244	0.1604	0.2076	0.3209	−0.0005
160	0.003761	0.1882	0.2559	0.3025	−0.0097
200	0.004252	0.2070	0.2980	0.2930	−0.0057
Neurosteroid (strength)					
0.50	0.007200	0.2626	0.5308	0.2613	−0.0242
0.55	0.006884	0.2569	0.5081	0.2668	−0.0212
0.60	0.006626	0.2516	0.4890	0.2663	−0.0210
0.65	0.006407	0.2473	0.4731	0.2657	−0.0180
0.70	0.006221	0.2441	0.4592	0.2676	−0.0223
0.75	0.006064	0.2409	0.4471	0.2635	−0.0138
0.80	0.005961	0.2392	0.4395	0.2689	−0.0217
0.85	0.005860	0.2371	0.4316	0.2707	−0.0212

Note. Seed = 42; baseline sparsity = 0.6 (activity pruning). Untreated reference: Persev. = 0.5247, Flex. = 0.9717, Acc. = 0.4972.

A.3 True Iso-Dose Matching

Treatment	Nearest param	Actual L1	Residual	In Tol.	Acute Persev.
Ketamine	regrow fraction = 0.1	0.005782	0.000782	True	0.2620
SSRI	epochs = 200	0.004252	0.000748	True	0.2930
Neurosteroid	strength = 0.85	0.005860	0.000860	True	0.2707

Metric	Ketamine	SSRI	Neurosteroid
Best Acute Persev.	0.2588	0.2930	0.2613
Best Relapse Δ	0.0316	−0.0097	−0.0242
Max Efficiency	45.4384	107.8521	43.3534
Mean Efficiency	36.4385	72.2164	40.5222

A.4 Multi-Seed Statistics

Metric	Ketamine	SSRI	Neurosteroid
Best Acute Persev.	0.2330 ± 0.0065	0.2831 ± 0.0099	0.2413 ± 0.0021
Mean Efficiency	35.2688 ± 8.1057	68.0495 ± 18.1388	36.4710 ± 8.4551

Best Relapse Δ	0.0799 ± 0.0379	0.0221 ± 0.0459	−0.0159 ± 0.0094

Note. Untreated perseveration across seeds = 0.5094 ± 0.0549.

A.5 Sensitivity and Ablation Battery

Sparsity	Untreated	Ketamine	SSRI	Neurosteroid
0.40	0.2627	0.2596	0.2623	0.2613
0.50	0.3476	0.2604	0.2655	0.2599
0.60	0.5247	0.2588	0.2930	0.2613
0.70	0.6218	0.2604	0.3631	0.3054

Configuration	Untreated Persev.	Ketamine Best	Ketamine Efficiency
GRU + modular	0.5247	0.2588	36.44
GRU − no modular	0.5043	0.2521	30.08
LSTM + modular	0.4815	0.2570	20.52
GRU + modular − no rec. bias	0.5247	0.2588	36.44

Switch interval	Input noise	Perseveration	Flexibility
30	0.8	0.4845	1.0383
50	0.8	0.4938	1.0375
50	1.2	0.5496	0.9744
80	0.8	0.6914	0.9872

Note. All results are from a hypothesis-generating in-silico model and are not validated mechanistic or clinical findings. Pruning is a computational proxy; iso-dose is a network-change convenience metric, not a pharmacological dose. Any treatment ordering holds only within this abstraction and must not be interpreted as evidence regarding ketamine, SSRIs, or neurosteroids in patients.
